# Flycatchers Copy Conspecifics in Nest-Site Selection but Neither Personal Experience nor Frequency of Tutors Have an Effect

**DOI:** 10.1371/journal.pone.0060395

**Published:** 2013-03-27

**Authors:** Tuomo Jaakkonen, Annemari Kari, Jukka T. Forsman

**Affiliations:** Department of Biology, University of Oulu, Oulu, Finland; Arizona State University, United States of America

## Abstract

Using the behavior of others in guiding one's own behavior is a common strategy in animals. The prevailing theory predicts that young age and the inexperience of an individual are expected to increase the probability of adopting the behaviors of others. Also, the most common behavior in the population should be copied. Here, we tested the above predictions by examining social information use in the selection of nest-site features with a field experiment using a wild cavity nesting bird, the collared flycatcher (*Ficedula albicollis*). We used an experimental design in which geometric symbols depict nest-site features. By manipulating the apparent symbol choices of early settled individuals and monitoring the choices of later arriving birds, we can study social information use without bias from learned or innate preferences. Flycatchers were found to use social information in the selection of nest-site features, with about 60% of the population preferring the manipulated conspecific choices. However, age and experience as explanatory factors suggested by the social information use theory did not explain the choices. The present result, in concert with earlier similar experiments, implies that flycatchers may in some situations rely more on interspecific information in the selection of nest-site characteristics.

## Introduction

Optimal decisions in spatially and temporally variable environments require information about local conditions. Information can be acquired by personal assessment (i.e. asocial information) but it requires time and energy and is therefore costly [1], [2]. Reducing these costs might be possible by observing the behavior of other, presumably more knowledgeable, individuals [1]. Using the behavior of others as a source of information – social information – is a widespread strategy in the animal kingdom [3], and it has been shown to affect many important decisions such as mate choice [4], [5], foraging [6], dispersal and breeding site decisions [7], [8], and anti-predator strategies [9]. Hence, social information use is an integral part of animal behavior and interactions among individuals, enabling faster adaptation to varying conditions compared to genetic evolution [3], [10]. Social information use may even lead to maladaptive choices [11], which demonstrates how influential social information can be.

Theory of social information use predicts when copying the behaviors of others should be favored over asocial learning or choosing at random [1], [12]. Firstly, social information should be favored when asocial information is costly, uncertain or lacking. Secondly, the behavior of most successful, older, resident or otherwise more knowledgeable individuals are expected to be copied. Thirdly, the most common behavior in the population is often adopted [1] even though other behaviors would do equally well [13].

The collared flycatcher (*Ficedula albicollis*), a small migratory cavity-nesting bird, is an excellent model organism for studying the prevalence of social information use in natural settings. The current evidence demonstrates that flycatchers use both intra- and interspecific social information in many important decisions. They gather intraspecific social information during the breeding season (density and success of conspecifics) and use it in habitat selection decisions in the following year [7], [14]. Some evidence also exists about conspecific attraction in breeding habitat selection upon arrival from migration [15]. Moreover, flycatchers also use interspecific social information (i.e. breeding density and nest-site choices of resident tits, their competitors) in their breeding area [16], [17] and nest-site [8], [18], [19] decisions upon arrival from migration. Furthermore, flycatchers' interspecific information use seems to follow the predictions derived from the intraspecific context because they not only copy the choices of good quality tit tutors but also reject the choices of poor tutors [18], [19].

In this study, we experimentally tested whether flycatchers use intraspecific social information in the selection of nest-site features and whether it follows the predictions of the social information use theory. In birds, the choice of a nest-site feature is of great importance because it is an essential niche dimension, has strong effects on fitness and is under disruptive natural selection driven by nest predation [20], [21]. Nest-site feature preferences are plausibly partially genetically determined [22]. We used the experimental design introduced by Seppänen et al. [8], [18] in which white geometric symbols depict nest-site features. Using non-natural nest-site features allows us to control for any learned or genetic preferences. We tested how the frequency of information sources (conformity bias), age, and earlier experience of the observer (site-faithful vs. immigrant) affect the prevalence of intraspecific social information use. We predicted that if flycatchers use conspecific social copying in nest-site selection i) they copy nest-site features from their conspecifics and copying behavior should increase with the increasing frequency of tutors, and ii) younger and immigrant individuals are expected to copy conspecifics more often than older and site-faithful ones. In addition, by comparing the present results in an intraspecific context to those obtained from an interspecific context [8], [18], we can get insight into whether flycatchers rely differently on information stimuli coming from conspecifics vs. heterospecifics.

## Methods

The experiment was carried out in 2009 on 8 forest patches (5–8 ha) in an agricultural landscape on the island of Gotland, Sweden, in the Baltic Sea. We set up nest-boxes in pairs into forest patches. Within a pair boxes were attached to the same tree species, at the same height and facing either the same direction or diagonally each other. We attached 40–50 box-pairs to each forest patch. Boxes within a pair were 2–5 meters apart and box-pairs were from 20–30 meters apart, thus one box-pair comprises a territory for one flycatcher pair. We used white plastic geometric symbols (a circle and a triangle) attached around the entrance holes of the boxes as a novel and artificial nest-site feature. Each box-pair included both of the symbols which were randomized to the boxes. Using abstract symbols effectively removes bias from earlier experience or innate preferences on behavior and enables making strong inference [8], [23].

**Figure 1:**
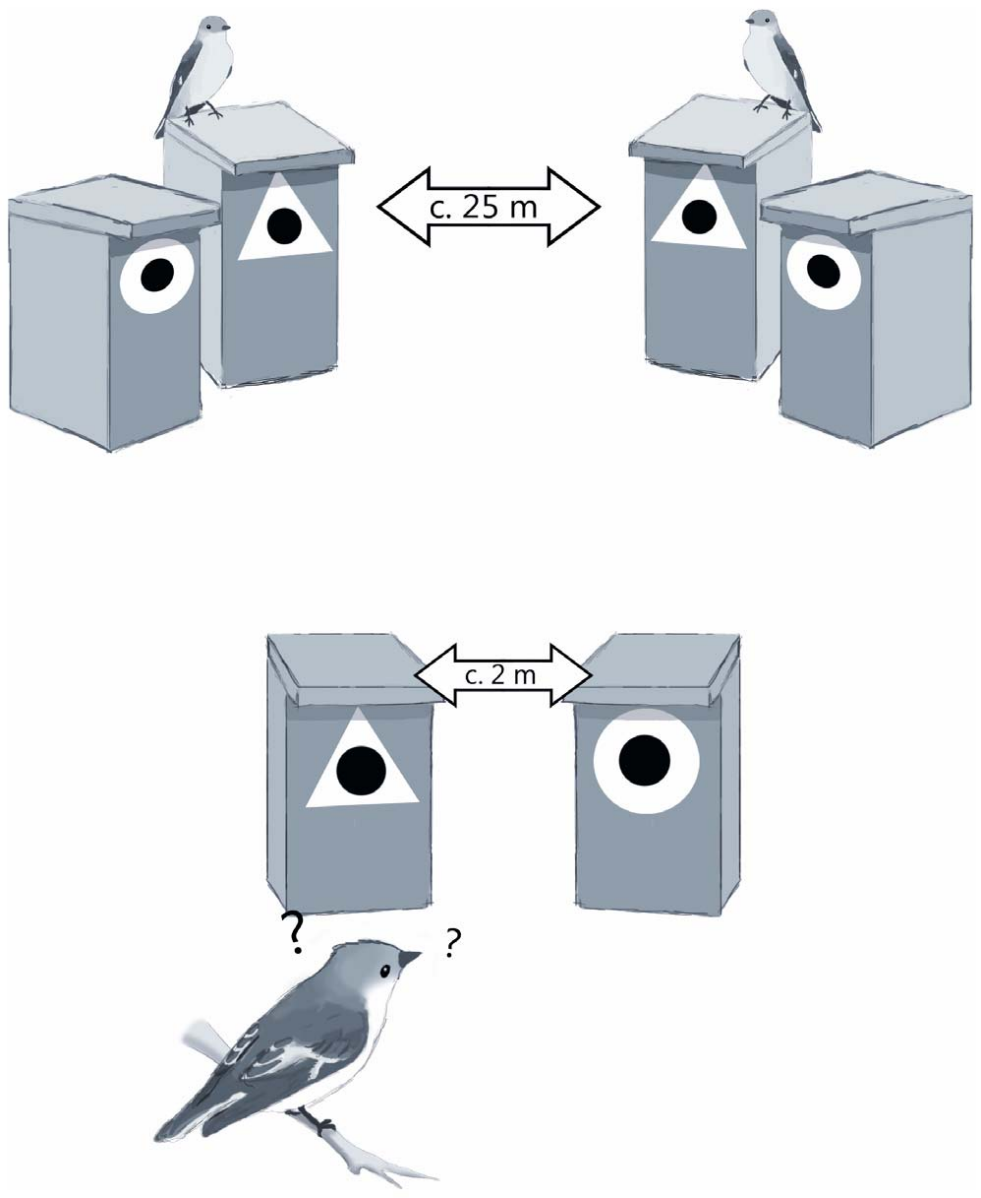
Experimental set-up. The first two flycatcher pairs were assigned to breed in a symbol that portrays the apparent choice of all conspecifics on the area (here a triangle) with an adjacent empty box with the other symbol (here a circle). Later arriving birds had to make a choice in an empty box-pair portraying apparent preferred and rejected symbols. Each later pair was also assigned the same manipulation symbol, thus increasing the amount of tutors for subsequent birds.

The first two flycatcher pairs in each area that initiated breeding were used to create the apparent nest-site feature preference of early settled conspecifics, *tutors* for subsequent birds, by attaching the same symbol on their boxes and the other symbol on the adjacent empty box. In four of the areas flycatchers were assigned to breed in a box with a triangle symbol and in the other four in a circle symbol. Assigning the symbol manipulation for an area was done randomly by a coin toss. Our experimental design (Figure 1) forces the subsequent birds to choose between two nest-boxes, otherwise similar but with a different white plastic symbol (for a detailed description of experimental design see [8]). This set-up applies the classical two-alternative forced-choice test widely used in psychology [24]. The choices of the first two model pairs were excluded from the data because they did not have any tutors. All the following choices were included in the data; therefore our data is representative of the whole flycatcher population, ranging from early arriving birds with only a few tutors to late arrivals with plenty of tutors.

The experimental set-up was finished about ten days prior to the arrival of the very first flycatchers and also before the nest building of most tits. Each box-pair was checked every second day during the whole arrival period of flycatchers from late April until the end of the spring migration in early June. Symbol choices were determined by the appearance of nest-material either in the circle or the triangle box in a box-pair. If flycatchers chose the conspecific symbol ( = ‘matching’), the choice and its date were recorded and the symbols in the box-pair were left untouched. If the non-conspecific symbol ( = ‘opposite’) was chosen, the choice and its date were recorded but the symbols were swapped within a box-pair. This was done to create the appearance for arriving birds that all the previously settled flycatcher females had preferred one symbol and rejected the other symbol. Each bird that started breeding in an area (choice recorded) was counted as a tutor for later breeding individuals (that made a choice two or more days later) on the area. This design allowed us to test the effect of the number of conspecific tutors on the use of social information. Symbols were removed from a box-pair if signs of occupancy by a tit pair were observed to exclude eligible social information of heterospecifics' nest-site features. Tits build their nest out of moss, hair and feathers, thus even early stages of a tit nest are easily recognizable from a flycatcher nest built from dry leaves and grass.

Adults were captured during breeding and their age [25] and site-faithful/immigrant status was determined. The information about status was acquired from rings of the birds because all birds that bred or were born in the areas in the previous year were ringed. Site-faithful individuals were second time breeders, or older, which were observed breeding on the same experimental area the year before. We did not observe any philopatric yearling females, which would have been born on their current breeding area the year before. Immigrants were new breeding birds to the area regardless of the age. In this study, neither birds nor bird nests were manipulated or sampled and therefore no ethics committee permit was required. Adult and young birds were handled and ringed under the ringing license from Swedish Museum of Natural History for professor Lars Gustafsson (University of Uppsala, Sweden). Hence, our study complied with the national legislation of Sweden concerning handling wild animals. Study areas are privately owned and permission to use the areas was acquired from the land owners.

Symbol choice data was first analyzed with a chi-square test including all eligible female choices to see if there is indication of conspecific copying in nest-site selection of the collared flycatcher. Only those birds' choices that initiated egg-laying were considered final and included in the analyses. The predictions of the social information use theory (see Introduction) were tested using the data that included female age and site-faithful/immigrant status using generalized linear mixed-effects models (GLMM) in R 2.13.1 [26]. The sample size for GLMMs was slightly smaller than in the whole data mainly because some females deserted their nests before they were captured.

The GLMMs were fitted with Laplace approximation as implemented in the R package ‘lme4’ (function ‘glmer’ [27]). Binomial error distribution with a logistic link function was utilized. Match of the chosen symbol with the tutor symbol (binary; match/mismatch) was the response variable. Fixed explanatory variables were the symbol (circle/triangle), the date of choice, number of tutors at the time of choice and experience variable which included site-faithful/immigrant status and age information. The experience variable was divided to three groups: yearlings born the previous year (all immigrants), older immigrant individuals, and older site-faithful individuals which bred on the same area the year before.

First we fitted a full model that included all possible main effects and interactions of the fixed explanatory variables, as well as random intercepts and slopes for different areas in relation to the number of tutors. The definitive model was obtained by reducing the fixed effects of the full model. Model selection was based on Akaike information criterion (AIC), but we also used visual evaluation of residual plots to assess model goodness-of-fit. Because p-values can be estimated too high in some cases when the error distribution is binomial [28], we conducted a permutation test to assess the reliability of p-values. We permutated the values of the response variable 10 000 times, and fitted the definitive model to the permutated data after each round of permutation. Point estimates of the fixed effects were saved after each round of model fitting and, consequently, we obtained empirical distributions of ten thousand estimates for the fixed effects. Finally, empirical p-values were calculated for each parameter by comparing the parameter estimates based on the actual data with the empirical distributions.

## Results

**Figure 2:**
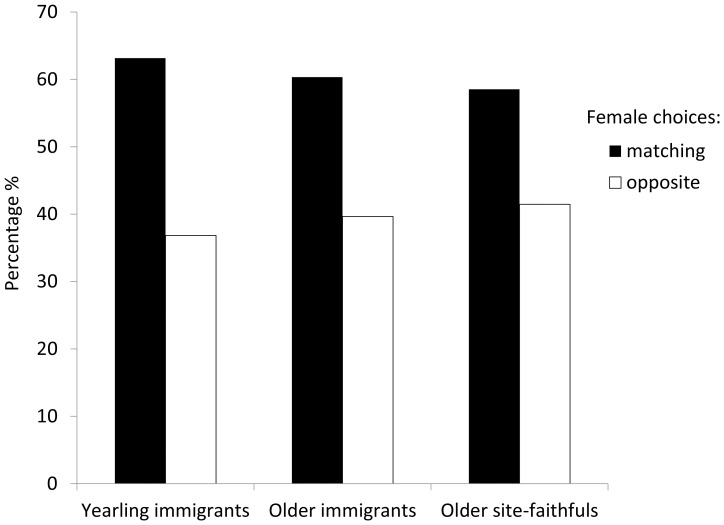
Flycatcher choices. The percentage of flycatcher females choosing matching (black bar) or opposite (white bar) symbol of the tutoring flycatchers in the three cohorts. Yearlings are individuals born the previous year, and are all immigrants (63.2% matching choices, n = 19). Older immigrants are older individuals which are new to the area (60.3.% matching choices, n = 58). Older site-faithfuls are older individuals, which bred on the same area the year before (58.5% matching choices, n = 41).

The data consisted of 135 symbol choices, and the number of breeding collared flycatcher pairs per study area (n = 8) varied from 12 to 27. In general, 59.3% of the females chose the conspecific (“matching”) symbol suggesting that flycatchers have a tendency to copy conspecific choices in nest-site selection (χ^2^ = 4.630, df = 1, two-tailed P = 0.031; we observed 80 matching choices and 55 opposite choices, and the expected is 50% for both groups). On circle manipulation areas 57.9% of choices (n = 69) were matching while on triangle manipulation areas 60.6% were matching (n = 66), suggesting that symbols *per se* did not affect female choices (Fisher's exact test two-tailed P = 0.861; in circle areas 40 choices matching, 29 opposite and in triangle areas 40 matching, 26 opposite). Furthermore, copying behaviour between cohorts was similar (Figure 2): in immigrant yearlings 63.2% (n = 19) of individuals copied the conspecific symbol, while in older immigrants 60.3% (n = 58), and in older site-faithful individuals 58.5% (n = 41) did so. Median choice date, as to be expected, was earliest for older site-faithfuls, in between for older immigrants, and latest for yearlings, nevertheless, choice dates between the cohorts mostly overlapped (first choice 1^st^, 1^st^ and 6^th^, median choice date 10^th^, 12^th^ and 14^th^, and last choice 24^th^, 24^th^ and 24^th^ of May for older site-faithfuls, older immigrants, and yearlings, respectively).

A GLMM analysis was done for choices that included female age and site-faithful/immigrant status information (n = 118). The best model with lowest AIC-value was in a GLMM with random area-specific slopes for tutor number which only included intercept and individual experience, both non-significant (Table 1). Permutation test confirms the results. GLMM analysis was also run with age and site-faithful/immigrant status as two separate binary variables which gave similar results (results not shown). Date of choice had no effect, and the prevalence of copying did not increase during the season or with the density of tutors and the experience of the individual had no effect.

**Table 1:** The best generalized linear mixed-effects models (GLMMs).

Model	AIC	Parameter	Estimate	SE	z	P value	P (perm.)*
1^A^	166.5	Intercept (Yearling)	0.535	0.477	1.121	0.262	0.403
		Site-faithful adult	−0.192	0.572	−0.335	0.738	0.386
		Immigrant adult	−0.116	0.547	−0.212	0.832	0.418
2^B^	170.5	Intercept (Yearling)	0.535	0.477	1.121	0.262	
		Site-faithful adult	−0.192	0.572	−0.335	0.738	
		Immigrant adult	−0.116	0.547	−0.212	0.832	

A) In 'lme4' syntax: match/mismatch of choice ∼ experience + (tutor number −1 | area)

B) In 'lme4' syntax: match/mismatch of choice ∼ experience + (tutor number | area)

* P value from permutation test, see Methods for details

Estimated fixed effects for experience variable of the two best GLMMs explaining the probability of flycatchers to copy the tutors' symbol choice. The models were fitted with Laplace approximation (R function ‘glmer’), binomial error distribution and a logistic link function. Only area-specific random slopes in relation to the number of tutors at the time of choice were included in model 1, whereas both area-specific random slopes and intercepts in relation to the number of tutors at the time of choice were included in model 2.

## Discussion

Our results suggest that flycatchers have a tendency to copy the choices of their conspecifics in nest-site selection; about 60% of the flycatcher females preferred the manipulated conspecific choices across the settlement period. However, our results did not support any of the predictions derived from the theory of social information use. Younger individuals and individuals with no or little experience about local ambient conditions are expected to rely more on social cues than older and experienced individuals [1], [12]. Neither age (breeding for the first time or an adult bird) nor site-faithful/immigrant status (female bred on the area in the earlier year or not) significantly explained the choices. Moreover, one of the most general predictions in social information use posits that copying should increase with the increasing frequency of tutors [1]. We did not find increasing preference of conspecific choices with the progress of settlement period and increasing number of tutors.

What could explain our results that do not support most predictions of the social information use theory? In some cases the correlation between behavioral strategy and social information use might be a by-product of another process. A recent study demonstrated with mathematical models [29] that division into behavioral roles in a producer-scrounger game can be simply the result of a sequential arrival. Arrival order can drive individuals to consistent roles in the use of social information. In their simulations first arrivals tended to implement the producer strategy and later arrivals were more prone to scrounge, and these behavioral strategies persisted in the individuals' further choices. Our results do not coincide with the theory of Dubois et al. [29] because arrival order did not affect social information use pattern in the experiment. However, in support for arrival order theory Seppänen and Forsman [8] showed in a two-species system that first arriving flycatchers use personal information and/or choose randomly in the selection of nest-site features, while about 75% (more than in our intraspecific study) of the late arriving birds relied on the manipulated apparent choices of resident heterospecifics, the tits, even though the number of earlier breeding tit tutors was about the same for all flycatchers.

The different context (intra- vs. interspecific) may explain the contrasting results between the present study and that of Seppänen and Forsman [8] and why we did not find support for our general predictions. Morand-Ferron et al. [30] showed experimentally that bird individuals can be consistent in their strategy use over a long period in producer-scrounger and patch-choice contexts, but only within one context while there was no behavioral correlation across the two different contexts. They found that context-specificity is an important part of within-individual behavioral plasticity. The results by Morand-Ferron et al. [30] cannot be directly applied in our study because we examined solely among-individual variation in behavior. However, it is possible that the flycatchers can also differentiate intra- and interspecific contexts and implement different social information use strategies accordingly; some flycatcher individuals may prefer social cues acquired from tits while some prefer conspecific cues. The results that neither age, earlier experience [cf.1] nor arrival time [cf.8,29] of flycatchers explained copying behavior support this possibility.

A likely explanation for potential differences between the use of intra- and interspecific social information use is the quality of information. Resident tits initiate breeding about two weeks earlier than flycatchers and tits most likely know the ambient environmental conditions better than recently arrived conspecifics. In addition, flycatchers have been found to judge the value of the tit tutors and their choices by tits' clutch sizes [18], [19], [31]. This information is more readily available and reliable for late than early arriving flycatchers. Tit clutch size information is not available or final for the first arriving flycatchers, because tits cover their eggs during egg laying period when their clutch is incomplete. Quantitative information provided by tits and their clutch size may explain the temporal effect in interspecific social information use of the flycatchers [8], instead of the suggested flycatchers' individual experience. In contrast, synchronously breeding conspecific tutors portray only qualitative presence-absence information in their choices. This could explain the more constant pattern of social information use in the present intraspecific experiment compared to the interspecific one [8]. Finally, flycatchers have been shown to also use intraspecific quantitative social information in their immigration decisions based on the reproductive success of conspecifics in the previous year [7], [32]. These examples imply that whether the acquired social information is quantitative or qualitative may impact flycatchers' decisions.

To conclude, the present result in concert with the earlier studies with similar experimental procedures reveal a rather fine-tuned decision-making system of the collared flycatcher. Our result suggests that flycatchers use conspecific social cues in nest-site selection but utilizing interspecific information from tits is more frequent in late breeding individuals [8]. This gives support for the Seppänen et al. [33] hypothesis that in some cases interspecific social information use can be more important than intraspecific information. Whether flycatcher individuals use social information differently from con- and hererospecific tutors (within individual variation) when both information sources are available simultaneously or if some individuals rely more on intraspecific and others interspecific information (between individual variation) is still unclear. More research on this topic is needed as individuals usually live in multispecies communities and amidst a continuous information flow from con- and heterospecific neighbors.
